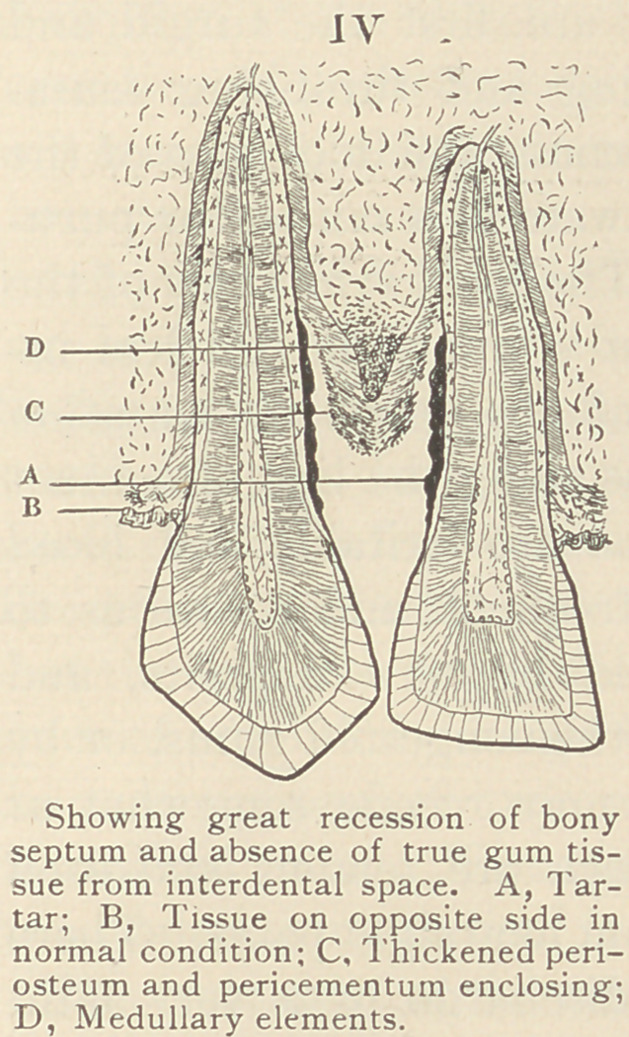# Pyorrhœa Alveolaris

**Published:** 1886-12

**Authors:** Alfred R. Starr

**Affiliations:** New York City


### Independent Practitioner.

Vol. VII. December, 1886. No. 12.

### Original Communications.

Note.—No paper published or to be published in another journal will be accepted for this department. All papers must be in the hands of the Editor before the first day of the month preceding that in which they are expected to appear. Extra copies will be furnished to each contributor of an accepted original article, and reprints, in pamphlet form, may be had at the cost of the paper, press-work and binding, if ordered when the manuscript is forwarded. The Editor and Publishers are not responsible for the opinions expressed by contributors. The journal is issued promptly, on the first day of each month.

### PYORRHŒA ALVEOLARIS.

Lecture before the Class of the New York College of Dentistry, Session of '85 and '86.

BY ALFRED R. STARR, M. D., D. D. S., NEW YORK CITY.

(Concluded from page 405)

The pathological changes in pyorrhœa are mainly such as may be readily seen or appreciated by the observer, and hence may also be classed as objective symptoms. In the acute form, or that due to the presence of foreign bodies (other than deposits of tartar), we have swelling and inflammation of the gums, ulceration of the gums and pericementum, exudation of pus, absorption of the alveolus, loosening of the teeth, etc. The process is usually a rapid one, and the symptoms so severe as to require immediate treatment. Owing to this fact the acute variety seldom makes much headway, for when the cause is removed the disease rapidly subsides. It is possible, though hardly probable, that a foreign body in the alveolus might excite so little irritation and remain so long as to cause a subacute inflammation of the pericementum, the formation of sanguinary calculus and the development of a chronic form from an acute case. I have known misplaced wedges of rubber, wood, or cotton to work down into the alveoli and excite an inflammatory action closely simulating true alveolar abscess. In a doubtful case I have even drilled into a tooth which presented the appearance of having a devitalized pulp (owing to its naturally dark color being increased by the presence of several amalgam fillings), and, on getting through the filling, struck sensitive dentine. Of course this was a signal to stop. The cold water test then proved that the sensitiveness was normal, and not due to pressure. On looking around for the cause of the abscess, I found in the alveolus of the affected tooth, or well down between it and the adjoining one, a small rubber wedge, which had been inserted two or three weeks previously, and was supposed to have fallen out. It produced only slight irritation at first, but soon developed marked inflammation and abscess.

The pathological changes in the idiopathic or chronic form are as follows: The first indication met with is a slight thickening or tumefaction of the margins of the gums, and this may be followed by nodular growths or spongy excrescences. There is a solution of continuity between the gums and the teeth, and then between the pericementum and the teeth. The separation of the pericementum begins at some portion of the neck of the tooth and extends gradually toward the apex. According to some of those who believe in the constitutional origin of this disease, the ulceration and separation of the pericementum is supposed to be due to an atrophic dyspepsia of the connective tissue; according to those who favor the germ theory, this phenomenon is attributed to the inroads of fungi. Other theorists attribute it to the irritation caused by salivary calculus; still others ascribe it to the result of primary or secondary catarrhal inflammation of the gums. With the exception, perhaps, of the last mentioned theory, it is possible that any one, or all, of these conditions may be instrumental in causing the characteristic lesion of this disease. I do not believe, for several reasons, that the process is really a catarrhal inflammation *per se.*

1st.—Because the tissue for which the disease seems to have a special predilection and upon which it exhibits its chief characteristics is not the mucous membrane, but the *pericementum.*

2nd.—Because the inflammatory process is so localized, and ulceration and discharge occur only at the points of connection with the teeth, and not upon the free surface of the mucous membrane. True, these parts are the ones subjected to the greatest amount of irritation, but the point I wish to make is, that we often have the same amount of irritation and apparently the same inflammation of the gums in cases of simple salivary calculus, without having the characteristic lesion of pyorrhœa alveolaris.

3rd.—Because the periosteum of the bone itself (outside of the sockets) is not involved.

4th.—Because we never have the deposition of lime-salts on the alveoli.

5th.—Because we so seldom, if ever, have necrosis of bone as a result. I know that this latter statement conflicts with the teachings of most of those who have written upon the subject; but in my experience I can only recall one case in which I could detect the presence of dead bone. Even in that instance I was in doubt as to whether the necrosis was the result of this disease, or whether there had been an abscess to cause it. If we had necrosis we would have bare bone and final exfoliation, but I think you will rarely, if ever, find such conditions as a result of pyorrhœa alveolaris *alone.* I believe the removal of the alveolus is done by a process of absorption, and not of necrosis. If you will examine a case in which the disease is well advanced, and pass a blunt excavator between the gum and the tooth, you will find that the gum is still adherent to the surfaces of the alveolar process; or if the gum be removed from between the teeth, that the fibrous covering of the bone still remains, and you will also find that the pericementum has not been detached from the remaining portion of the alveolar wall. If the source of nutrient supply was from the tooth to the alveolus, or if the lime-salts were deposited upon the alveoli instead of upon the teeth, then we would expect to find the alveoli necrosed; but we know that the opposite conditions prevail, and in the great majority of cases we will find that the fibrous covering of the alveolus is *not* removed, and the bone is *not* exposed or necrosed. I believe the pathological changes are, essentially, the following:

Assuming that we have a local irritation resulting in ulceration of the gums as a starting point, and that there exists a constitutional tendency to the disease, this irritation extends to the pericementum, and the osteoblasts, which form a layer next to the cementum, are incited to cause the deposition of lime-salts. This deposit serves to increase the irritation and inflammation, and the neighboring cells (osteoblasts) are also involved. The pericementum is thus gradually removed from the tooth by an ulcerative process, but is not detached from the alveolus. (See Fig. I.) That portion of the alveolus, thus covered by pericementum which has been detached from the tooth, no longer having any function to perform in giving nourishment and support to the tooth (through the intervention of the pericementum), is absorbed. This absorption, I think, occurs in the same manner, and for the same reason as when the tooth is extracted.

I

Section through canine and lateral, showing conditions supposed to exist in the earlier stages of the disease. A, Tartar; B, Tissues at neck of tooth in normal condition; C, Gum slightly receded; D, Pericementum detached from the tooth, but not from the alveolus; E, Medullary tissue.

The fibrous tissue of the gums remains in contact with the alveolar processes, or the periosteal covering with which it is normally united. The gums collapse and recede as the alveoli are absorbed. The gums do not, however, recede to an extent corresponding with the amount of absorption of the socket. While the bone recedes, I believe the gums and pericementum approximate as the medullary tissue between them is removed. (See Fig. II.) If we examine a case in which the disease attacks the proximal surfaces of the roots, in many instances we will find that, while the bony septum is much absorbed and has receded to quite an extent, there is still a fibrous

II

Section of upper canine. Tissues normal on palatal aspect. Pyorrhœa alveolaris on labial side. A, Tartar; B, Gum which has receded slightly; C, Pericementum separated from the tooth and presenting an ulcerating surface; D, Medullary elements of the receding bone.

partition between the teeth, connected on the one hand with the gum, and on the other with the alveolar septum, which partition is probably made up of the two layers of hypertrophied pericementum, between which we will find the medullary tissue into which the bone is changed as the lime-salts are removed.

III

Section, through canine and lateral, showing absorption of bony septum, leaving bridge of gum tissue in interdental space. A, Tissue at neck in normal condition; B, Bridge of gum tissue cut horizontally; C, Tartar; D, Pericementum—thickened and inflamed—separated from tooth, but still adherent to alveolus; E, Medullary tissue.

The ulcerative process may cut through this fibrous partition, and we then have a sort of bridge of gum tissue in the interdental space. (See Fig. III.) This bridge of gum tissue may atrophy or ulcerate, and we then have, on the outer and inner aspects of the teeth, an irregular line of gum, having thickened margins and sending no prolongations between the teeth. When in this condition the gums frequently present spongy excrescences or nodular prolongations at the points opposite the interdental spaces. If we could examine such a case microscopically, we would expect to find the component parts of an interdental septum to be such as are depicted in Figure IV, the periosteum and the two layers of pericementum enclosing the medullary elements of the receding bone. In very acute cases the ulcerative process may result in exposure of the bone, but this is the exception rather than the rule.

IV

Showing great recession of bony septum and absence of true gum tissue from interdental space. A, Tartar; B, Tissue on opposite side in normal condition; C, Thickened periosteum and pericementum enclosing; D, Medullary elements.

It is said that we may have localized dissolution of the pericementum and the formation of calcular material in enclosed pockets, but this is still a matter of controversy. In some cases the irritation of the calcular material on the roots (which, according to Prof. Abbott, is always present in this form of the disease) may excite a cellulitis in the neighboring gum tissue, and the formation of a real gum-boil or false alveolar abscess results.

Pockets are formed between the gums and the teeth by the ulceration of the pericementum and absorption of the alveoli, in which pockets food is liable to lodge and increase the irritation. Saliva is also apt to get into these pockets by capillary attraction, and depositing its lime-salts, increases the calcular accretions and intensifies the disease. There is some discharge from these pockets; first serous, then purulent, and finally sanious or ichorous.

We may have secondary catarrhal affections of the antrum or nose. The disease may spread from the alveolus of one tooth to the adjoining ones. We may have separate teeth affected on opposite sides of the mouth, or on the same side, and the intervening teeth may be free from its ravages; while in other cases all the teeth may be affected. It is rather the exception than the rule to have the entire circumference of a root involved. Assuming the root to be quadrilateral, we will usually find that it is affected on one side, or two or three at the most.

The subjective symptoms, or those appreciable to the patient, may be classed as local and constitutional. In the acute or traumatic form the local symptoms predominate, the disease being usually of such short duration that general symptoms rarely supervene. The local symptoms of the acute form are, first the turgid and irritable feeling of the gums, pain, swelling and throbbing sensation when an abscess is developing, tenderness and looseness of the adjoining teeth, and the patient may be aware of a sanious or purulent discharge from the seat of injury. The local symptoms of the idiopathic or chronic form are somewhat similar, though less intense. There is the same turgid feeling of the gums or sensation of fullness, and there may be some redness along the line of contact with the necks of the teeth. The gums are irritable and bleed easily, and the hemorrhages may be so frequent and severe as to excite alarm. The soreness is increased by new deposits, and diminished by occasional depletion from the congested gums, or by the removal of the deposits. The gums may recede somewhat as the disease progresses. The affected teeth are tender, and soon become loose. The patient may suffer from neuralgic pains. There may be a profuse sanious or ichorous discharge of a very fetid odor. The absorption of the alveoli frequently results in gradual displacement of the teeth and marked deformity. The disease may progress very slowly, affecting first one tooth and then another, of all the teeth may be affected at the same time. These chronic cases, in some instances, may last for quite a number of years before resulting in the loss of the tooth or teeth.

If the disease be severe and long-continued, and especially if many teeth are affected, general symptoms are apt to supervene. In such cases there may be loss of appetite and disturbances of the whole digestive apparatus. The patient may have indigestion, coated tongue, foul breath, acid eructations and diarrhœa. He becomes debilitated, and may have headaches, neuralgic pains in the eyes and ears, melancholia or hypochondriasis.

The diagnosis of the acute form is not difficult except when we have the condition simulating true alveolar abscess. In such cases we have to determine whether the affected teeth are alive or not. If they are alive and we can find no tartar on the roots to account for the disturbance, we must make search for foreign bodies, projecting fillings, or any source of irritation.

The diagnosis of the idiopathic or chronic form is not difficult with the exception, again, of those cases in which we have the formation of false abscess. In ordinary cases the congested gums, slight recession of the gums, separation of the pericementum and absorption of the alveoli, the presence of sanguinary calculus, the formation of pockets, loosening of the teeth, the exudation of pus, or a sanious discharge on pressure, etc., will give ample evidence of the nature of the trouble. In those cases in which we have the development of false abscess, we must use great care in differentiating between this and the ordinary variety of alveolar abscess. Notice carefully the color of the tooth or teeth, use the electric mouth lamp in doubtful cases, apply the cold or hot water test, and search carefully for the presence of tartar or other foreign bodies. The presence of tartar on the roots would not be a sure indication that the case was one of false abscess, but its absence and the absence of any other foreign body would indicate pretty clearly that the case was one of true alveolar abscess, due to the presence of a devitalized pulp. We must be very confident of our diagnosis before undertaking radical treatment.

The treatment of the traumatic form is mainly local, the disease being usually of such short duration that constitutional treatment is rarely required. The indications are to open the abscesses if there be any, remove deposits or foreign bodies, syringe well with antiseptics, and leave the rest to nature.

In the idiopathic or chronic form the treatment is both local and constitutional. Dr. Rawls says: "There is but one principle upon which all local treatment should be based, viz., that new tissue will not grow upon dead tissue. In other words, broken-down nutrient continuity must have protection against substances inimical to the establishment of embryonic tissue." The first thing to be accomplished is a thorough removal of all foreign materials from the teeth, as far as the pericementum has become detached.

In those very rare cases in which we have necrosis of the alveolar processes, remove as much of the devitalized portion as you can. During the operation, and after removing the deposits and getting the roots as smooth as possible, syringe the pockets with an antiseptic solution. For this purpose we may use carbolic acid (1-100), peroxide of hydrogen (diluted one-half), tartrate of chinoline (1-40), listerine (pure or diluted to one-half or one-third), chloride or sulphate of zinc (grs. X to ℥ i), or aromatic sulphuric acid (pure or diluted one-half). I have found a combination of tannin and glycerine (tannin ʒ i and glycerine ℥ i) of some value as an astringent dressing for the pockets. I am indebted to Prof. Abbott for the following prescriptions, which will be found very useful in the treatment of this disease:

| | ℞ | |
|---|---|---|
| No. 1. | Morphia, Sulphat, | gr. iv. |
| | Acid, Carbolic. | |
| | Acid, Tannic, | *aa* ʒ ss |
| | Glycerina. | |
| | Aqua, Distil. | *aa* ℥ ss |

M.

*Sig.*—Apply on a pledget of cotton to the diseased parts, or use with a syringe or spray apparatus.

| | ℞ | |
|---|---|---|
| No. 2. | Acid, Carbolic. | |
| | Acid, Tannic, | *aa* ʒ ss |
| | Glycerina. | |
| | Aqua, Distil. | *aa* ℥ ss |

M.

*Sig.*—Same as for No. 1.

℞

| | | |
|---|---|---|
| No. 3. | Acid, Carbolic. | |
| | Acid, Tannic. | |
| | Tinct. Iodin. | *aa* ʒ ss |
| | Glycerina. | |
| | Aqua, Distil. | *aa* ℥ ss |

M.

*Sig.*—Same as for No. 1.

Number one is to be used when there is much pain attending the inflammatory process; number two, when we wish simply an astringent and antiseptic effect; and number three, when we want a more stimulating effect, as may be necessary in long-standing or intractable cases. In cases where we have much loss of connection between the tooth and the socket, with some lime deposit, it has been recommended to use a solution of aqua regia (nitro-hydrochloric acid) in the proportion of one part of the acid to seven of water; and in cases of still greater loss of attachment, with loss of considerable portions of the alveolar plates, the same authorities advise the use of a caustic paste, made by melting together caustic potash (potassa fusa) and crystallized carbolic acid, the object being to produce a scab or eschar, which will act as a protective covering for the protoplasm which is to effect repair. I have had no experience in the use of these powerful escharotics in the treatment of this disease, and, therefore, cannot speak from my own knowledge for or against their clinical value; but if it be true, as many assert, that the milder means are equally efficacious, I should hesitate to use such powerful remedies. If we desire a caustic effect, a moderately strong solution of chloride of zinc (20 or 30 grs. to ℥ i), in ordinary cases, is sufficiently escharotic to form a protective coating for the new material, to destroy the infusoria, and to modify the inflammatory process. In obstinate cases we may use a solution of sixty grains to the ounce, or even stronger. The zinc chloride is rendered still more acceptable in these cases, from the fact that it produces a slough which is in itself inodorous. Since the chloride of zinc seems to meet these requirements so well, where is the necessity for using a remedy more penetrating, more diffusive, and withal so difficult to manipulate as is the caustic paste above referred to?

If the teeth are much loosened, trim them down so that they will not strike the opposing ones when the jaws are at rest, and support them with ligatures or artificial appliances, so as to protect the parts from violent motion. Endeavor to make them as immovable as possible. In bad cases, I believe this precaution is very necessary. Slight stimulation, by friction of the gums or by medicaments, may frequently be necessary to establish a healthy circulation, but constant or violent motion of the loosened teeth must necessarily be detrimental to the reparative process. Cleanliness is very essential, hence the patient should be carefully instructed as to the manner in which he should brush and care for his teeth. Astringent washes are frequently of value. The use of chewing gum is advocated by some, as tending to induce cleanliness of the teeth and a healthy condition of their surroundings. After removing the deposit, in most cases all that is necessary is to syringe the parts carefully with a mild antiseptic solution, and then follow up the case to see that the pockets are kept in as clean and aseptic a condition as possible. I sometimes instruct my patients how to syringe the pockets, and let them wash out the parts two or three times a day with a solution of carbolic acid (1 to 100), using care not to disturb the granulations. In cases in which the pockets are quite deep and much difficulty is experienced in cleaning the roots and keeping them clean, it has been recommended to slit up the pockets to their extremities, and then treat as an open wound. A system of sponge grafting has been tried in cases where there has been much loss of the surroundings of the teeth, and good results are claimed for this method of treatment.

When the deposit on the root is very adherent and extends almost or quite to the apex, it has been recommended to extract the tooth if it be an anterior one, cleanse the outside and the canal thoroughly, trim the end of the root slightly, fill the canal, and replant. Some operators claim great success for this mode of treatment, and say that with this method they have obtained complete reproduction of tissue, even when there had been removal of the socket almost to the apex of the root. I do not think this method is likely to become very popular, for the following reasons :

It is only applicable to the anterior teeth, and such teeth can usually be pretty thoroughly scraped and cleaned while in the mouth, unless the disease is well advanced and the pockets or sinuses are tortuous.

If resorted to in advanced cases where the teeth are quite loose, the death of the pulp and of portions of the cementum militates against the success of the operation.

I have performed replantation for the cure of pyorrhœa in only one instance, and in that case the disease was so far advanced, the tooth was so loose and so much covered with the deposit, that the pulp and the greater portion of the cementum were devitalized. The tooth, a left upper central, was so loose and tender that the patient could not stand the operation of cleansing it in the mouth. Knowing I could do nothing with it as it was, I proposed this operation to the patient, telling him frankly that the chances of success were very small, but that there was a faint hope of improving the condition of affairs. I soon gained his consent to try the operation. The tooth was extracted, its outer surface cleaned carefully, the pulp canal cleaned and filled, the end of the root trimmed slightly, both tooth and socket treated antiseptically, when it was replaced and held in position with ligatures. The pockets were syringed and treated with antiseptics about every other day during a period of two weeks. After the first day or two there was no pain, except on pressure, and no appreciable discharge. At the end of two weeks the ligatures were removed, and it was found that the portion of cementum which was still alive had united with the tissues, but so large a portion of the cementum was devitalized before the operation was performed that the union was not extensive enough to keep the tooth firm. It was left for a week without any support, when it was found that it dropped gradually until it came in contact with its antagonist. I reapplied the ligatures a few days ago, and still have them on. When I last saw the patient, I could not detect the slightest discharge, and the parts looked quite healthy, except for an increased redness of the gum. The tooth is somewhat firmer than before the operation, but it is still so loose that it will never be of much service in mastication.*

There are cases in which the operation of replantation would seem to be justifiable and likely to succeed. For example, we sometimes meet with instances in which the pockets will extend in a spiral direction toward the apex, beginning, perhaps, on the an-

* This operation was not a success. After about two weeks the ligatures came off again, and had been off but a few days, when the patient, in pressing and pulling on the tooth (presumably to test its firmness), forced it out of the socket. It may have been that it was kept in place more by atmospheric pressure and the elasticity of the soft tissues than by any actual union.

terior or labial surface and extending around to the palatal, with no external opening for the sinus on the palatal surface of the tooth. Obviously, such a tooth could not be cleaned without resorting to extensive cutting of soft and hard tissue to get at the pockets, and in such a case, if ordinary means failed, I think I would resort to this operation.

If we use any of the acid preparations for an astringent or caustic effect, we must be careful to protect the crowns of the teeth, and to wash them off with an alkaline solution (such as bicarbonate of soda), after using the remedy.

The general or constitutional treatment consists in the use of proper diet, out-door exercise and general supportive treatment. In cases where the secretions of the mouth have become much vitiated, or where the existence of a purulent discharge has so much interfered with the functions of digestion and nutrition as to produce anorexia or general debility, tonics may be required. In such cases we may prescribe preparations of iron, quinine, or any of the bitter tonics, and advise the use of cod-liver oil. Of the iron preparations, I would prefer the dialysed or the reduced iron, because these forms are non-irritating, non-constipating, and do not directly affect the teeth. The dialysed iron may be given in doses of ten or fifteen drops, and the reduced iron in doses of two grains after meals. Sulphate of cinchonidia has been advised as a tonic in these cases, because it is believed that this substance is readily converted into the cruorin or hæmoglobin of the blood. In cases where the disease is associated with gout, rheumatism or diabetes, endeavor to combat the general disorder.

PROPHYLAXIS.—Since salivary calculus is supposed to be the most common exciting cause of this disease, in order to avoid this deposit and prevent the occurrence or recurrence of pyorrhœa alveolaris, we should advise cleanliness, proper brushing, care and exercise of the teeth. We should see that partial artificial dentures do not bear too heavily on the teeth or irritate the gum-margins. Regulate the diet, and insist upon the patient's indulging in healthful exercise. Instruct the patient to exercise the teeth properly in mastication.

The prognosis of the acute or traumatic form is good. The retention of the teeth is accomplished, their vitality preserved and the disease cured on removal of the cause. The prognosis of the idiopathic or chronic form is not so good. In regard to this form of the disease, Prof. Abbott says "pyorrhœa alveolaris is never cured; i. e., the normal condition of the parts restored." He also states that it can be relieved for the time being, and may appear to be cured, but the condition that first developed the disease remains in the system in spite of treatment, and that the same results will recur, unless the case is followed up and treatment repeated every few months—certainly as often as once or twice a year. When due to the presence of salivary calculus, the disease can be cured, or rather the parts can be restored to a healthy condition, provided the sanguinary deposit on the roots can be entirely removed. In cases in which the disease has made little progress and there has been but slight ulceration of the pericementum, I believe the parts may be restored to a normal condition, with the exception, perhaps, of a slight recession of the gums, due to cicatrization of the new tissue. It is claimed by some writers that the protoplasm is capable of being transformed into the tissues normal to the parts, even to the reproduction of new bony sockets for the teeth. I have seen cases of simple salivary calculus in which this new formation of osseous tissue has apparently occurred, and where teeth, deprived of a greater portion of their sockets and very loose, have become as firm and their surroundings to all appearances as perfect as before. Whether or not there was complete restoration in these cases, even to the formation of new bone and new pericementum, I cannot say positively, but it certainly seemed as if such was the case. It is quite possible that we may have a restoration of bone in pyorrhœa alveolaris also, for the bone is very seldom deprived of its periosteal covering in this disease; but the pericementum (toward the neck of the tooth) is to a certain extent destroyed by the ulcerative process, and whether it can be reproduced and again attach itself to the scraped surface of the cementum, is still a matter of doubt. I think it more likely for the reproduction of the bony socket to occur in cases of salivary calculus proper, where the absorption of bone is due to the pressure of a foreign body, than in cases of pyorrhœa alveolaris, where the process (so far as the absorption of bone is concerned) is more of a physiological one, in which the bone atrophies and is absorbed because it has no further function to perform. Without doubt, we sometimes see a formation of new tissue in cases of pyorrhœa alveolaris. The gum may return almost to its normal position and the teeth become firm, but may it not be that this firmness is due to a return of the irritated, inflamed and thickened pericementum to a normal condition, and to the formation of fibrous or scar tissue to take the place of the lost pericementum and alveolus? I believe that in the majority of cases this is the condition which prevails, and that we do not have a complete restoration of the parts to their normal condition, particularly in those cases which are far advanced. Probably the reason we do not have this reproduction is because of the difficulty in keeping the teeth in a proper state of fixation. We must not despair of a good result because we do not happen to see marked improvement in a short time. A good rule is to keep up the treatment until suppuration ceases, and the production of new tissue begins, or until there is no hope of such a favorable result. It may require weeks and even months of careful treatment in order to restore the parts to a good, healthy condition.

## Figures and Tables

**I f1:**
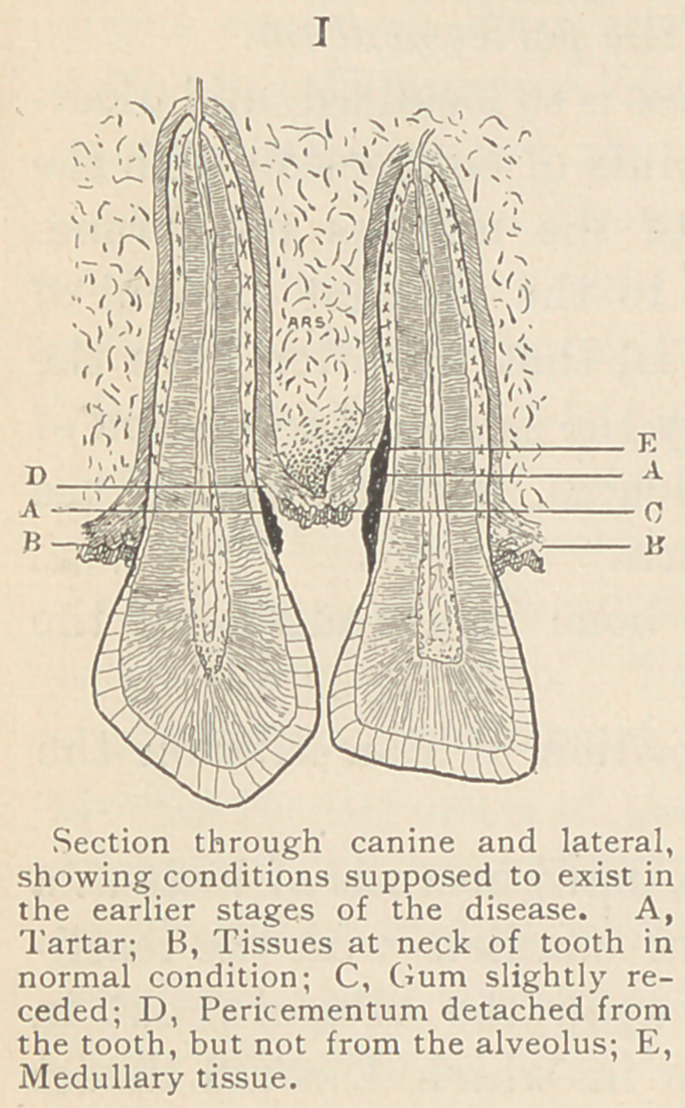


**II f2:**
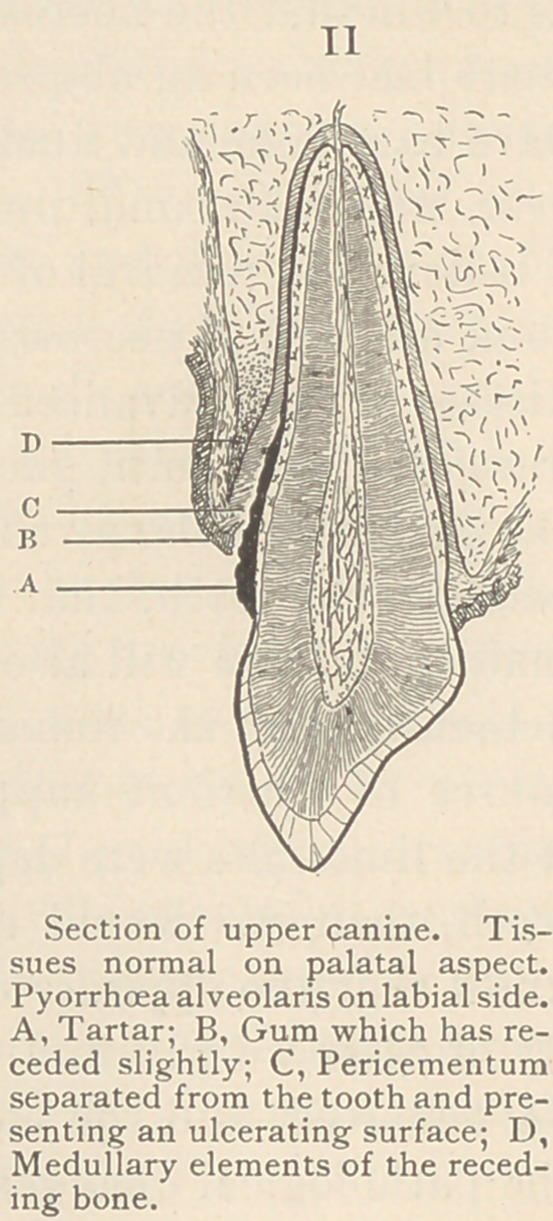


**III f3:**
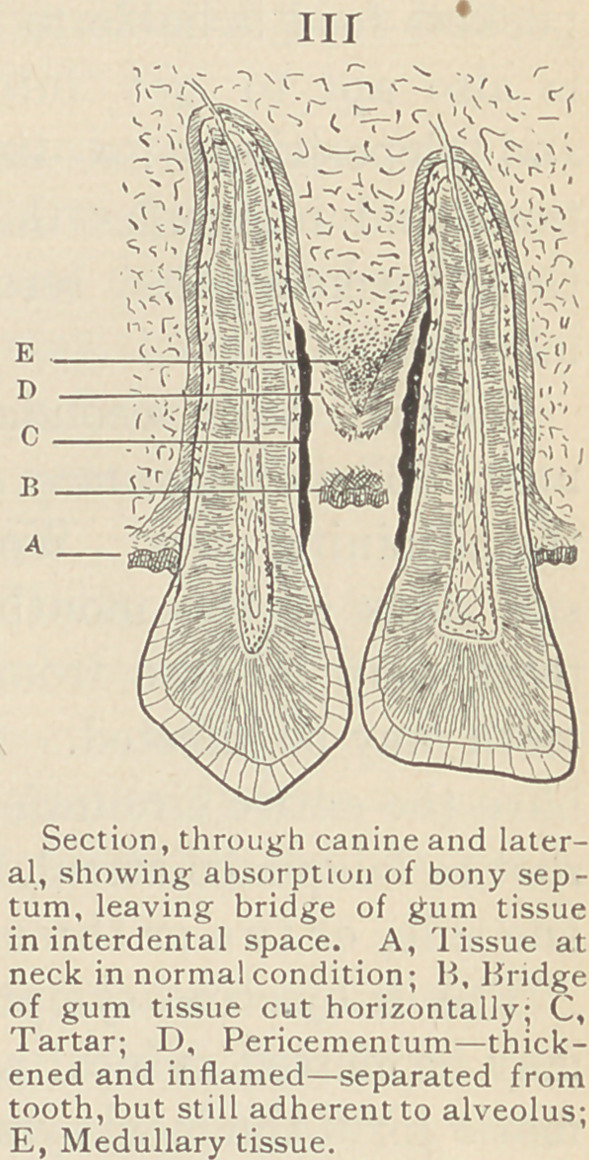


**IV f4:**